# CMR-assessed aortic arch stiffness is associated with brain tissue integrity assessed by diffusion tensor imaging in patients with hypertension

**DOI:** 10.1186/1532-429X-17-S1-P401

**Published:** 2015-02-03

**Authors:** Michiel Sala, Annette van den Berg - Huysmans, Jeroen van der Grond, Anne Brandts, Albert de Roos, Jos J Westenberg

**Affiliations:** 1 Leiden University Medical Center, Leiden, Netherlands

## Background

Increased aortic stiffness may lead to insufficient flow wave dampening and subsequent transmission of excessive pulsatile energy towards end-organs such as the brain. It has been shown that CMR-assessed aortic stiffness may augment cerebral small vessel disease in patients with hypertension, as assessed by conventional structural magnetic resonance imaging (MRI). However, in addition to these overt brain abnormalities, currently it is unknown whether aortic stiffening relates to subtle changes in brain tissue integrity, which may be a precursor to overt brain abnormalities. Diffusion tensor imaging (DTI) in the brain has been used to evaluate such subtle changes in tissue integrity. The aim of this study was to assess the association between aortic arch pulse wave velocity (PWV) as a marker of arterial stiffness and brain changes assessed by conventional structural MRI as well as DTI in patients with hypertension.

## Methods

**Figure 1:**
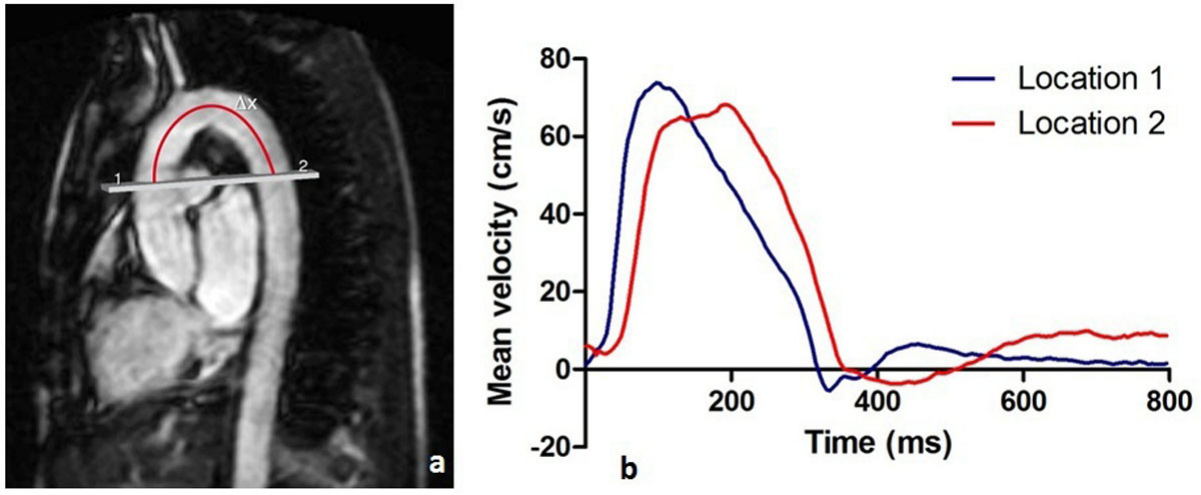
**Aortic arch pulse wave velocity assessment: (A) Double-oblique parasagittal image of the thoracic aorta.** The gray line represents the acquisition plane for one-directional through-plane velocity-encoded MRI which is positioned perpendicular to the ascending aorta (1) and additionally transects the proximal descending aorta (2). Pulse wave velocity (PWV) is determined from the propagation of the blood flow velocity, defined from the velocity-time curves recorded at locations 1 and 2 (B). PWV is defined as Δx/Δt, with Δx the path length along the centerline of the aorta from positions 1 to 2 and Δt the transit time for the foot of the velocity wave to propagate from positions 1 to 2.

78 patients with hypertension (35 men, mean age 46 ± 1 years) were prospectively included. Aortic imaging was performed using 1.5T MRI. To assess PWV over the aortic arch, one-directional through-plane velocity-encoded MRI was performed, planned perpendicular to the ascending aorta and additionally transecting the proximal descending aorta (Figure 1). Brain MRI was performed on 3.0 T MRI. Linear regression analysis was performed to assess the association between aortic arch PWV and brain macrostructure (brain volume and white matter lesion volume) and microstructure (fractional anisotropy [FA], mean diffusivity [MD], axial diffusivity [AxD], and radial diffusivity [RD] (Pierpaoli et al. Neuroimage 2001)). Models were adjusted for several cardiovascular risk factors.

## Results

Aortic arch PWV was not associated with brain macrostructure. In contrast, aortic arch PWV was associated with changes in white matter (FA: β = -0.30, p = 0.011; MD: β = 0.31; p = 0.005; AxD: β = 0.24, p = 0.040; RD: β = 0.33; p = 0.002) and grey matter integrity (MD: β = 0.28, p = 0.006; AxD: β = 0.27, p = 0.012; RD: β = 0.29, p = 0.006) (Table 1). This effect was independent of age, gender, body mass index, smoking, mean arterial blood pressure, duration of hypertension and cerebrovascular disease.

**Table 1:** Association between CMR-assessed aortic arch PWV and brain volume, white matter lesion volume, and DTI measures of brain microstructure.

Brain volume (cm^2^)	β	P-value
White matter	-0.22	0.094

Grey matter	-0.18	0.061

**Ln white matter lesion volume (mL)**	0.05	0.713

**FA**		

White matter	-0.30	0.011*

Grey matter	0.01	0.938

**MD**		

White matter	0.31	0.005*

Grey matter	0.28	0.006*

**AxD**		

White matter	0.24	0.040*

Grey matter	0.27	0.012*

**RD**		

Grey matter	0.33	0.002*

White matter	0.29	0.006*

P-values are from linear regression analysis, adjusting for age, gender, body mass index, smoking, mean arterial blood pressure, duration of hypertension and cerebrovascular disease. FA: fractional anisotropy; MD: mean diffusivity; AxD: axial diffusivity; RD: and radial diffusivity. * P-value < 0.05.

## Conclusions

Our data suggest that aortic arch stiffness is independently associated with changes in brain tissue integrity in patients with hypertension. Subtle changes in brain microstructure are related to increased stiffness of the aortic arch, even in absence of overt brain abnormalities.

## Funding

N/A.

